# Supplementary material for: Modeling glioblastoma heterogeneity as a dynamic network of cell states
Source: Mol Syst Biol. 2021 Sep 16;17(9):e10105. doi: 10.15252/msb.202010105 (PMC8444284; doi:10.15252/msb.202010105)
Supplement: Supplementary file 5 — Source Data for Figure 3 [file MSB-17-e10105-s001.zip › Figure3A_sourcedata/GSEA_3065/hallmarks_state4.GseaPreranked.1623416296068/HALLMARK_UNFOLDED_PROTEIN_RESPONSE.html]

Details for gene set HALLMARK\_UNFOLDED\_PROTEIN\_RESPONSE[GSEA]

|  || Dataset | state4 |
| Phenotype | NoPhenotypeAvailable |
| Upregulated in class | na\_neg |
| GeneSet | HALLMARK\_UNFOLDED\_PROTEIN\_RESPONSE |
| Enrichment Score (ES) | -0.52004105 |
| Normalized Enrichment Score (NES) | -1.7636286 |
| Nominal p-value | 0.0 |
| FDR q-value | 0.004908586 |
| FWER p-Value | 0.038 |
Table: GSEA Results Summary

  

Fig 1: Enrichment plot: HALLMARK\_UNFOLDED\_PROTEIN\_RESPONSE      
 Profile of the Running ES Score & Positions of GeneSet Members on the Rank Ordered List

  

| PROBE | GENE SYMBOL | GENE\_TITLE | RANK IN GENE LIST | RANK METRIC SCORE | RUNNING ES | CORE ENRICHMENT || 1 | TUBB2A |  |  | 61 | 0.287 | 0.0252 | No |
| 2 | RPS14 |  |  | 178 | 0.211 | 0.0324 | No |
| 3 | DDIT4 |  |  | 493 | 0.138 | -0.0014 | No |
| 4 | ATP6V0D1 |  |  | 635 | 0.122 | -0.0092 | No |
| 5 | PAIP1 |  |  | 643 | 0.121 | 0.0044 | No |
| 6 | EEF2 |  |  | 729 | 0.110 | 0.0042 | No |
| 7 | LSM1 |  |  | 1090 | 0.083 | -0.0438 | No |
| 8 | EIF4A2 |  |  | 1311 | 0.070 | -0.0707 | No |
| 9 | CNOT2 |  |  | 1479 | 0.063 | -0.0900 | No |
| 10 | TTC37 |  |  | 1486 | 0.062 | -0.0833 | No |
| 11 | DCTN1 |  |  | 1550 | 0.060 | -0.0862 | No |
| 12 | SEC31A |  |  | 1815 | 0.052 | -0.1224 | No |
| 13 | YWHAZ |  |  | 1896 | 0.050 | -0.1293 | No |
| 14 | POP4 |  |  | 1955 | 0.048 | -0.1328 | No |
| 15 | CNOT6 |  |  | 2037 | 0.046 | -0.1402 | No |
| 16 | EXOSC4 |  |  | 2038 | 0.046 | -0.1346 | No |
| 17 | PSAT1 |  |  | 2153 | 0.043 | -0.1478 | No |
| 18 | NHP2 |  |  | 2254 | 0.040 | -0.1590 | No |
| 19 | CEBPB |  |  | 2420 | 0.037 | -0.1811 | No |
| 20 | BANF1 |  |  | 2472 | 0.035 | -0.1850 | No |
| 21 | SERP1 |  |  | 2633 | 0.032 | -0.2069 | No |
| 22 | YIF1A |  |  | 2723 | 0.030 | -0.2176 | No |
| 23 | TATDN2 |  |  | 2999 | 0.025 | -0.2590 | No |
| 24 | ATF4 |  |  | 3132 | 0.022 | -0.2775 | No |
| 25 | IMP3 |  |  | 3198 | 0.021 | -0.2855 | No |
| 26 | WIPI1 |  |  | 3310 | 0.018 | -0.3012 | No |
| 27 | CNOT4 |  |  | 3536 | 0.011 | -0.3362 | No |
| 28 | DCP1A |  |  | 3620 | 0.008 | -0.3486 | No |
| 29 | GOSR2 |  |  | 3729 | 0.004 | -0.3655 | No |
| 30 | VEGFA |  |  | 3787 | 0.001 | -0.3746 | No |
| 31 | EIF4E |  |  | 3810 | 0.000 | -0.3781 | No |
| 32 | DNAJB9 |  |  | 3929 | -0.006 | -0.3964 | No |
| 33 | HERPUD1 |  |  | 3930 | -0.006 | -0.3957 | No |
| 34 | EXOSC5 |  |  | 4150 | -0.018 | -0.4288 | No |
| 35 | ATF6 |  |  | 4204 | -0.022 | -0.4347 | No |
| 36 | ATF3 |  |  | 4262 | -0.026 | -0.4407 | No |
| 37 | HSPA9 |  |  | 4311 | -0.030 | -0.4448 | No |
| 38 | KHSRP |  |  | 4351 | -0.032 | -0.4472 | No |
| 39 | SSR1 |  |  | 4490 | -0.044 | -0.4641 | No |
| 40 | EXOSC2 |  |  | 4767 | -0.065 | -0.5006 | No |
| 41 | HYOU1 |  |  | 4825 | -0.069 | -0.5013 | No |
| 42 | ERO1A |  |  | 4850 | -0.072 | -0.4964 | No |
| 43 | FKBP14 |  |  | 4869 | -0.073 | -0.4904 | No |
| 44 | EIF4A3 |  |  | 4899 | -0.075 | -0.4859 | No |
| 45 | EIF4G1 |  |  | 5092 | -0.091 | -0.5057 | No |
| 46 | STC2 |  |  | 5150 | -0.097 | -0.5031 | No |
| 47 | MTHFD2 |  |  | 5256 | -0.107 | -0.5070 | Yes |
| 48 | DCP2 |  |  | 5283 | -0.108 | -0.4979 | Yes |
| 49 | DDX10 |  |  | 5349 | -0.114 | -0.4945 | Yes |
| 50 | XBP1 |  |  | 5441 | -0.123 | -0.4942 | Yes |
| 51 | DKC1 |  |  | 5452 | -0.125 | -0.4805 | Yes |
| 52 | KIF5B |  |  | 5464 | -0.127 | -0.4668 | Yes |
| 53 | SLC7A5 |  |  | 5488 | -0.131 | -0.4545 | Yes |
| 54 | EIF4A1 |  |  | 5506 | -0.132 | -0.4411 | Yes |
| 55 | SRPRA |  |  | 5602 | -0.145 | -0.4387 | Yes |
| 56 | EXOSC9 |  |  | 5643 | -0.151 | -0.4266 | Yes |
| 57 | LSM4 |  |  | 5673 | -0.155 | -0.4123 | Yes |
| 58 | EIF4EBP1 |  |  | 5747 | -0.171 | -0.4031 | Yes |
| 59 | DNAJC3 |  |  | 5774 | -0.178 | -0.3855 | Yes |
| 60 | SPCS1 |  |  | 5857 | -0.196 | -0.3747 | Yes |
| 61 | NPM1 |  |  | 5884 | -0.203 | -0.3541 | Yes |
| 62 | NOP56 |  |  | 5921 | -0.213 | -0.3338 | Yes |
| 63 | SEC11A |  |  | 5933 | -0.216 | -0.3091 | Yes |
| 64 | SPCS3 |  |  | 5948 | -0.220 | -0.2845 | Yes |
| 65 | SRPRB |  |  | 5958 | -0.225 | -0.2583 | Yes |
| 66 | HSPA5 |  |  | 6061 | -0.274 | -0.2412 | Yes |
| 67 | CALR |  |  | 6153 | -0.344 | -0.2138 | Yes |
| 68 | FUS |  |  | 6170 | -0.371 | -0.1710 | Yes |
| 69 | PDIA6 |  |  | 6213 | -0.482 | -0.1187 | Yes |
| 70 | HSP90B1 |  |  | 6225 | -0.509 | -0.0581 | Yes |
| 71 | CKS1B |  |  | 6234 | -0.523 | 0.0047 | Yes |
Table: GSEA details [plain text format]

  

Fig 2: HALLMARK\_UNFOLDED\_PROTEIN\_RESPONSE: Random ES distribution      
 Gene set null distribution of ES for **HALLMARK\_UNFOLDED\_PROTEIN\_RESPONSE**
